# Vaccine Passport and Traveler Behaviors in the New Market of the Domestic and International Tourism Industry Facing the With-Corona Era

**DOI:** 10.3389/fpsyg.2022.900976

**Published:** 2022-06-10

**Authors:** Lanji Quan, Amr Al-Ansi, Antonio Ariza-Montes, Marcelo Arraño-Muñoz, Gabriele Giorgi, Heesup Han

**Affiliations:** ^1^College of Hospitality and Tourism Management, Sejong University, Seoul, South Korea; ^2^Faculty of Hospitality and Tourism Management, Macau University of Science and Technology, Macao, Macao SAR, China; ^3^Social Matters Research Group, Universidad Loyola Andalucía, Córdoba, Spain; ^4^Facultad de Ciencias Sociales, Universidad Autónoma de Chile, Santiago, Chile; ^5^Department of Human Science, European University of Rome, Rome, Italy

**Keywords:** vaccine passports, vaccine certificates, perceived usefulness, destination trust, risk perception, incentives, international tourism, tourism entrepreneurs

## Abstract

To ensure a smooth and rapid recovery of tourism, countries around the world are stepping up vaccinations against COVID-19. China, in particular has a very high vaccination rate due to its own vaccine production. Following this trend, many countries have started introducing vaccine passports as an alternative solution to verify valid and vaccinated travelers. This study attempted to understand the fundamental perceptions of travelers’ intentions using vaccine passports. A total of 601 samples were investigated and analyzed. As a result, four factors were identified: perceived usefulness, destination trust, risk perception, and perception of incentives. Also, this study performed means comparisons analysis with the major demographic characteristics of respondents. Based on this study, it is expected that the results will contribute to the revival of the travel industry in the future and provide valuable implications for marketing plans to help the travel industry suffer from COVID-19.

## Introduction

China’s tourism industry has been one of the world’s most in-demand inbound and outbound tourism markets since the early 1980s, and has also contributed significantly to the Chinese economy. In 2019, the domestic travel population reached 6 billion, an exponential increase compared to the number of trips to China a decade ago (Ma, 2020). However, the tourism sector has been hit hard by the unpredictable spread of COVID-19, and it is unknown when the tourism industry will be completely recovered (Chung et al., 2021; Liu-Lastres et al., 2021). Therefore, governments and other organizations around the world are developing vaccine passports to enable greater mobility and access to other services for people vaccinated against COVID-19 to accelerate social resumption and return to daily life (Tanner and Flood, 2021). The vaccine passports/certificates are currently being seen as the only way to revive international travel and are starting to be used in several countries. The “Vaccine Passport” is a certificate that demonstrates if a person has been vaccinated against the coronavirus (BBC News, 2021). Seira (2021) reports that vaccine passports/certificates will be a matter of convenience, not mandatory, as it is useful, timely, and informative. The introduction of vaccine passports will not only boost the travel industry (Shaikh et al., 2021), but will also encourage more people to get vaccinated (Guidi et al., 2021). Vaccine passports will be widely used in the travel industry and other large gatherings such as sporting events, experts say (Walker, 2021; Mithani et al., 2022). China had achieved immunization of 86.73% of the population by 12May 2022 (Our World in Data, 2022). Vaccination rates for residents of large cities (Beijing, Shanghai, etc.), front-line workers, medical workers, and delivery workers have exceeded 90% (Nature, 2021). Chinese authorities declare a strict “zero-COVID” policy, which severely disrupts daily life and halts economic activities (VOA News, 2022). Nevertheless, until 2021, the Chinese authorities were under full control of the COVID-19, but after the 2022 Olympics, the COVID-19 is spreading again. As the population is large, the authorities are judging that Chinese people cannot go “live with COVID-19” like other countries. However, since economic growth is also important, the tourism industry is expected to reopen soon.

A significant investment is required to introduce new systems such as vaccine passports. However, the potential benefits of vaccine passports cannot be achieved unless travelers appreciate the system. Users’ expectations and attitudes toward vaccine passports may differ. For example, there are people who travel in pursuit of 100% safety, and others who want to travel to safe places as much as possible and prefer to use a system such as a vaccine passport (Volgger et al., 2021).

The use of vaccine passports is an unavoidable phenomenon and is already being used in many countries (e.g., China, Turkey, Malta, Saint Kitts, Greece, Australia, Singapore, South Korea, New Zealand, EU countries, the United Kingdom, the United States etc.). Vaccinations are not compulsory, but outdoor activities are restricted if not vaccinated in some countries such as France (Euronews, 2021). Vaccine passports are being introduced in different ways from country to country, allowing tourists to fill out and print information online or generate a QR code to use (European Commission, 2021). The form required by country and city is different, but individuals can receive a QR code by filling out simple vaccination information on the self-portal website. However, there are some concerns such as the risk of using vaccine passports, destination trust by using vaccine passports, and perceived usefulness of using vaccine passports (Matsuura, 2021). In April, the World Health Organization (WHO) suggested that “immune passports” could actually increase the risk of continued transmission because people holding immunized passports would ignore public health recommendations on physical distancing (WHO, 2021a). Moreover, according to a study by Olliaro (2021), the COVID-19 vaccine does not effectively immunize the patient 100%. Thus, it can be predicted that travelers may have a higher risk perception and a lower willingness to travel during an epidemic.

Although there are many studies on the use of vaccine passports in the tourism industry, studies on consumers’ awareness of vaccine passports are lacking (Shin et al., 2021; Mithani et al., 2022). Thus, this study extracted people’s perception of vaccine passports/certificates into four factors. The first factor is the perceived usefulness of using vaccine passports was investigated. Numerous studies (e.g., Rivera et al., 2015; Upadhyay et al., 2018) have found that usefulness has a positive influence on attitudes and behavior intention. The second factor is destination trust in using vaccine passports. After the COVID-19 crisis, many tourist destinations have lost trust. In particular, many people do not visit places where there are many confirmed cases of COVID-19 because they perceive tourist destinations as a risk factor. The third factor is risk perception in using vaccine passports. Many studies show that using vaccine passports causes some risks, but in some form, it is inevitable that it will be widely implemented over a period of time as an interim recommendation during a pandemic (e.g., Baylis and Kofler, 2021; Pavli and Maltezou, 2021). In a statement on 5 February 2021, WHO laid down its reasons for not supporting vaccine certificates, based on ethical, legal, scientific, and technological reasons (WHO, 2021b). WHO recommends that people who are vaccinated should continue to comply with other risk reduction measures when traveling (WHO, 2021a). The last factor is the perception of incentives provided by the tourism industry to vaccinators. Many companies in the tourism industry (hotels, airlines, restaurants, travel destinations, etc.) offer various incentives to encourage vaccination. Although many events in the tourism industry are used as marketing tools to increase revenue, it is rare to work with governments to provide incentives to motivate customers to vaccinate. According to Koo et al. (2020), incentives play a vital role in motivating people, so it can be seen as an optimal means of facilitating traveler behavior. Therefore, this study explored travelers’ attitudes and travel intentions for Chinese based on potential factors on the perception of vaccine passports use. By identifying key factors in tourist perception of vaccine passports use, managers will provide better service and lead to successful marketing.

## Literature Review

### Perceived Usefulness of Vaccine Passports/Certificates

Perceived usefulness is defined as the degree/level to which people believe that using an object or nominated system would improve its performance, such as increasing processes speed, work performance, productivity efficiency, and making work easier and more useful (Davis, 1989, 1993). Huang et al. (2017) has identified perceived usefulness as the prospective value when using a certain structure of a system. Research by Chang and Chen (2020) has empirically assured the significant positive interactions and association between perceived usefulness and satisfaction of individuals. Cho et al. (2020) denoted that usefulness is a critical element influencing changes in consumer behavior. In tourism research, usefulness is often used as a measure to understand travelers’ intentions to adopt new technologies. Kim and Lee (2014) found that perceived usefulness plays as a positive motivator that helps generate intent to use, and individuals are more likely to continue using the system when it brings usability or usefulness. Vaccine passports are a newly introduced system for revitalizing the travel industry. Vaccine passports can be downloaded online, but platforms vary by country. In addition, vaccine passports grant preferential conditions to certain privileges, such as concerts, restaurants and indoor gathering places (Thomas et al., 2021). Based on this potential usefulness of vaccine passports in this study, we hypothesize that users can maximize the benefits of this new system to revive the travel industry and change the lives of people suffering from the COVID-19. The following hypotheses statements were established below:

H1: The usefulness of vaccine passports/certificates has an important and positive impact on tourists’ attitudes toward international travel.

H2: The usefulness of vaccine passports/certificates has an important and positive impact on tourists’ attitudes toward domestic travel.

### Destination Trust

Trust is known as a focal predictor in hospitality and tourism studies due to its competence to strengthen the relationship between stakeholders (Artigas et al., 2017). In other words, it has pragmatic attributes to increase/decrease interactions between two or more parties. In the tourism literature, scholars have actively assessed the internal and external characteristics of destination trust (Artigas et al., 2017; Al-Ansi and Han, 2019). Accordingly, Sirdeshmukh et al. (2002) reported that destination trust is usually formulated as the volume of trust and certainty gained by a traveler about a service provider of a specific object at a tourist site as a relationship exchange between two parties. Jeaheng et al. (2020) indicated that trust is a substantial prerequisite for travelers to choose a tourist destination. Thus, the tourism and hospitality players rely on constructing some solid partnerships between the destination practitioners and tourists (Kim et al., 2011; Jeaheng et al., 2020). Furthermore, destination trust has a significant influence on the psychological mood of tourists where it leads to magnify the travel intention (Wenping and Chunxiao, 2014). The critical role of trust increases magnificently during health crises. Recently, the WHO (2021b) has declared six predictors of trust that fall during the COVID-19 period namely (competence; objectivity; fairness; consistency; sincerity; faith). Slovic (1993) pointed out that once trust is lost, it is difficult to re-establish. As it is extensively known that trust plays an important role in forming tourist satisfaction and loyalty to a destination (Orth and Green, 2009; Kim et al., 2011; Artigas et al., 2017), the goal of this study is to continuously build destination trust by using vaccine passports. Tourists are widely preferred to visit places that they perceive to be trustworthy (Al-Ansi and Han, 2019). Accordingly, trust became an essential component for successfully running tourism marketing (Choi et al., 2016). Therefore, to ensure a healthy and safe environment, many authorities and industry players have expressed interest in demanding high immunization rates (Elejalde-Ruiz, 2020; Perobelli and Garcia, 2020). Hence, this study focused on the nexus between destination trust and traveler attitudes due to the use of vaccine passports to provide a knowledge base for the tourism market hit by the COVID-19.

H3: Destination trust due to the use of vaccine passports/certificates has an important and positive impact on tourists’ attitudes toward international travel.

H4: Destination trust due to the use of vaccine passports/certificates has an important and positive impact on tourists’ attitudes toward domestic travel.

### Risk Perception of Using Vaccine Passports/Certificates

Perceived risk was conceptually introduced to the study of consumer behavior in marketing by Bauer (1960). Its broad meaning covered different types of risk, such as performance, financial, environmental, healthy, physical, psychological, satisfaction, social, political instability, disease, and time risks (Simcock et al., 2006; Chen et al., 2017; Olya and Al-Ansi, 2018; Huang et al., 2020; Chua et al., 2021). According to Rittichainuwat and Chakraborty (2009), risk perception is enhanced when the safety and security of travelers is focused on infection with the infectious disease such as COVID-19. This also was investigated and affirmed by Chua et al. (2020). Perceived risk could therefore affect the overall travel plan, traveler behavior (Reichel et al., 2007; Chua et al., 2021), and future travel intention (Simcock et al., 2006; Simpson and Siguaw, 2008). The perceived risk significantly triggers traveling intention or selected a specific destination which in turn changed individual’s travel plans, travel to a particular place or avoid a certain destination (Matyas et al., 2011; Schroeder, 2015). Perceived risk not only affects decisions about where to travel, but also whether or not to travel (Lepp and Gibson, 2003; Reisinger and Mavondo, 2005; Rittichainuwat and Chakraborty, 2009; Olya and Al-Ansi, 2018). Tourists decide where to travel based on their perception of the destination rather than realistically. As perceived risk is the strongest predictor of travel avoidance (Cahyanto et al., 2016; Volgger et al., 2021), tourists are likely to make decisions based on their biased risk perception (Roehl and Fesenmaier, 1992; Chua et al., 2021). More and more countries have announced the use of vaccine certificates/passports to boost the tourism industry. This study focused on examining the effects of vaccine passports on risk perception, attitudes, and travel intentions. Therefore, the following hypotheses statements were established:

H5: Risk perception due to the use of vaccine passports/certificates has a significant and negative impact on tourists’ attitudes toward international travel.

H6: Risk perception due to the use of vaccine passports/certificates has a significant and negative impact on tourists’ attitudes toward domestic travel.

### Perception of Vaccine Incentives

By definition, an incentive is an external persuasion factor that encourages an individual’s motivation (Palmer, 2012). The government in China has offered a variety of incentives and remuneration, such as cash rewards, to encourage more residents and attract people to get COVID-19 vaccinations as they step up their mass vaccination campaigns (Wanqing, 2021). In particular, the tourism industry provides various incentives to vaccinated people and encourages citizens to vaccinate by using gift vouchers, cash vouchers, movie tickets, free flight ticket upgrades, and free drinks provided by the hotel as prizes (Wang, 2021). Such rewards and incentives are not exclusive practices to Chinese local authorities. While Russian (e.g., Moscow) attempted to attract more people by serving them with ice cream to encourage residents to get COVID-19 shots, restaurants in the UAE (e.g., Dubai) gave some discounts vouchers for those successfully vaccinated (GQ-News, 2021). In the United States, since vaccination programs began to be implemented, governments and private companies have been offering incentives such as tax cuts and free air tickets to entice those who hesitate to get vaccinated (Miriam Berger, 2021). In some famous hotels (The Shilla Hotel etc.) in Korea, early check-in and late check-out benefits are provided, and at buffet restaurants, 20% off up to 4 people and up to 82% off on accommodation vouchers are provided (Son Go-eun, 2021). In incentive research, studies on the role of incentives given by companies to employees and incentives granted by the government to companies are being actively researched (Groves, 1973; Gibbons, 1998; Glewwe et al., 2010; Carletti et al., 2021), but there are few studies in the travel industry that cooperate with the government to incentivize tourists to motivate. According to the study of Gneezy (2003), most economic theories are based on the proposition that incentives and performance are highly associated, but in some cases, introducing external incentives can change perceptions of activities and lower performance. Therefore, the following hypothetical statements were established focusing on the effects of incentives provided by the travel industry on travel attitudes.

H7: Incentives provided to vaccine passports holders in the tourism industry have an important and positive impact on tourists’ attitudes toward international travel.

H8: Incentives provided to vaccine passports holders in the tourism industry have an important and positive impact on tourists’ attitudes toward domestic travel.

### Attitude and Travel Intention

Attitude represents an individual’s overall assessment of behavioral performance. The attitude and behavior intention are intertwined concepts that discussed in several studies (Zarrad and Debabi, 2015; Jeaheng et al., 2020; Kim et al., 2021). Attitude toward behavior refers in a constant way to both responses (positive or negative) toward a particular behavior/object, such as service/product use, choice or design (Quintal et al., 2010; Han and Yoon, 2015). Moreover, tourist attitudes describe the psychological tendencies expressed by positive or negative assessment of travelers when engaging in particular behaviors (Ajzen, 1991; Kraus, 1995). The term “attitude toward the behavior” is commonly applied and adopted in socio-psychological concepts and rational choice decision-making models as it is a crucial theoretical concept in explicating human decisions processes and behaviors (Ajzen, 2001; Jeaheng et al., 2020). Therefore, attitude toward a behavior is a volitional attribute that produces and forms an intention (Perugini and Bagozzi, 2001). It is widely known that attitude triggers one’s behavioral intentions (Ajzen, 1991). To sum up, the more favorable the attitude toward the behavior, the stronger the individual’s will to the behavioral intention. Therefore, in this study, the following hypothetical statements were established to investigate the relationship between attitudes toward vaccine passports use and intention to travel domestically and abroad.

H9: Tourists’ attitude toward the use of vaccine passports/certificates has an important and positive impact on the intention to travel abroad.

H10: Tourists’ attitude toward the use of vaccine passports/certificates has an important and positive impact on the intention to travel domestically.

## Methodology

### Qualitative Approach for the Perception of Vaccine Passports/Certificates

In spite of many previous studies, in-depth interviews were conducted with 12 people (6 males and 6 females) for about a month in April 2021 to obtain more accurate perception factors related to the use of vaccine passports. The subjects included national officials, tourism management Master’s researchers and Doctoral researchers, travel agency staff, hotel staff, and travel lovers (travel once a year at least). Interviews were conducted online due to the coronavirus pandemic and lasted ~15 min per person, and participants were free to discuss and provide their opinions. Questions included *(1) What do you think about the introduction of vaccine passports during COVID-19? (2) How do you feel about using a vaccine passport when traveling, attending events or gatherings?* Through this qualitative approach through interviews, 11 items were derived, including convenience of vaccine passport use, sense of safety, low risk of infection, reduction of quarantine costs and time, trust in destinations, and use of various incentives (Airline, Hotel, Restaurant). Then, these 11 items were combined with 13 items derived from previous studies (Davis, 1993; Selnes and Sallis, 2003; Ngai et al., 2007; Olya and Han, 2020; Wang et al., 2021) and classified into four factors.

### The Questionnaire and Conceptual Framework

The survey of this study was developed into three parts: (1) perceptions of vaccine passports, (2) attitudes toward domestic and overseas travel intentions using vaccine passports, and (3) domestic and overseas travel intentions using vaccine passports. The study composition was rated on multiple items using a 7-point Likert scale ranging from ①extremely disagree to ⑦extremely agree. Perceived usefulness (6 items) was determined by Davis (1993) and Ngai et al. (2007) and modified according to the current study. For destination trust, 3 items were extracted based on the study of Selnes and Sallis (2003). In addition, risk perception of 6 items was extracted through the study of Olya and Han (2020). Perception of incentives questionnaire items was reviewed by Wang et al. (2021), 8 items were created according to the current research. Regarding the items related to the attitude toward travel intention toward the use of vaccine passports, 5 items were extracted, mainly from Kassem et al. (2003) and Jalilvand et al. (2012). Finally, travel intention (5 items and 3 items) was adopted and modified from Lam and Hsu (2006). The key purpose of this study is to investigate the effect of vaccine passports on tourist attitudes and the influence of tourist attitudes on domestic and international travel intentions during the COVID-19 period.

### Data Collection and Profiles of the Respondents

The questionnaire (Table 1) included questions about study structure, study description, and measures of demographic background. In order to improve the face validity, a total of 20 people received a preliminary questionnaire by e-mail. Based on their feedback, the correction and improvement were made on the questionnaire. The data was conducted with the Chinese population for about 40 days starting in June 2021 and was collected through the most trusted and popular survey company, Wenjuanxing online survey form. The sufficient explanations about vaccine passports were provided to the participants before filling out the questionnaire. The data were analyzed with SPSS Statistics 23.0 and AMOS program 22.0 and after removing 56 unusable responses (unavailable data: answered all questions with 1 or 7), a total of 601 usable cases have finally remained for data analysis. Among the 601 participants in the survey, 312 (51.9%) were male and 289 (48.1%) were female. The highest respondents’ age range was 30s (45.8%), and that was followed by the age range of 20s (37.3%), 40s (13.5%), 50s or older (3.3%) and 10s (0.2%). 41.6% of respondents reported that their monthly income was about 6,000–9,999 RMB, and 29.1% of respondents reported their wages were about 3,000–5,999 RMB. As for the status of marriage, married respondents accounted for 66.4% and singles accounted for 32.6%. The number of annual international travel, once a year visitors accounted for the most at 56.7%, followed by 2–5 visits with 29%. On the other hand, as for the number of annual domestic trips, 2–5 visitors accounted for 76.9%, followed by 6–10 visits per year with 16%.

**TABLE 1:** Profiles of the respondents (*N* = 601).

Variable	Category	Percentage (%)
Gender	Male Female	312 (51.9%) 289 (48.1%)
Age	10s (18–19) 20s 30s 40s 50s or older	1 (0.2%) 224 (37.3%) 275 (45.8%) 81 (13.5%) 20 (3.3%)
Educational level	High school degree or below Three-year college education Bachelor’s degree Master’s degree or above	17 (2.8%) 110 (18.3%) 354 (59.9%) 120 (20%)
Income	< 3,000 RMB 3,000–5,999 RMB 6,000–9,999 RMB > 10,000 RMB	35 (5.8%) 175 (29.1%) 250 (41.6%) 141 (23.5%)
Marriage	Single Married Others	196 (32.6%) 399 (66.4%) 6 (1%)
Average travel abroad/year	0 1 time 2–5 6–10 <10 times	84 (14%) 341 (56.7%) 174 (29%) 1 (0.2%) 1 (0.2%)
Average travel domestically/year	0 1 time 2–5 times 6–10 times <10 times	0 32 (5.3%) 462 (76.9%) 96 (16%) 11 (1.8%)

## Data Analysis and Results

### Exploratory Factor Analysis of the Measurement Model

Described in Table 2, we initially performed an exploratory factor analysis (EFA) using varimax rotation techniques to reveal the structure and dimensions of people’s perception of vaccine passports use in China. A total of 23 items were developed based on previous research and in-depth interview, and factors were derived. The Kaiser-Meyer-Olkin (KMO) value was 0.966 (*p* < 0.000), confirming the suitability of EFA. Eight identified constructs with eigenvalues >1 accounted for about 81.916% of the total variance. The coefficient values (factor 1 = 0.952, factor 2 = 0.876, factor 3 = 0.966, and factor 4 = 0.965) were all greater than cutoff standard of 0.70, indicative that all 4 identified constructs were adequate to proposed criteria of Nunnally and Bernstein (1978). Moreover, normality evaluation disclosed that skewness values ranging from (−0.964 to 1.664), and the kurtosis values ranging from (−0.360 to 1.925) which reported acceptable scores that adequately fell between (−2.00 and + 2.00), which implies that the current data set of our study was free from any skewness and kurtosis drawbacks.

**TABLE 2:** EFA result (*N* = 601).

Factors	Loading	Skewness	Kurtosis
**Factor 1** Perceived usefulness of vaccine passport Eigen-values 1.598; Variance Explained 19.024%; Cronbach’s alpha 0.952			
1. It is convenient to use when the vaccine passport/certificate comes out as a paper or digital format.	0.747	−0.915 (0.100)	0.237 (0.199)
2. Using vaccine passport/certificate when traveling is helpful as it reduces separate testing for COVID-19.	0.714	−0.627 (0.100)	−0.360 (0.199)
3. Vaccine passport/certificate gives me faster access to travel without a two-week quarantine.	0.758	−0.785 (0.100)	−0.111 (0.199)
4. Vaccine passport/certificate use improves the quality of travel.	0.719	−0.747 (0.100)	−0.253 (0.199)
5. Vaccine passport/certificate will be useful during traveling.	0.753	−0.838 (0.100)	0.012 (0.199)
6. Using the vaccine passport/certificate will be fit my needs.	0.750	−0.846 (0.100)	−0.063 (0.199)

**Factor 2** Destination trust for vaccine passport Eigen-values 1.089; Variance Explained 11.273%; Cronbach’s alpha.876			
1. I believe that destinations that accept vaccinated travelers will provide high quality and efficient tourism services.	0.790	−0.754 (0.100)	−0.217 (0.199)
2. I can rely on destinations that accept travelers with vaccine passport/certificate.	0.800	−0.761 (0.100)	−0.114 (0.199)
3. I believe that destinations that accept vaccine passport/certificate holders will be honest and sincere in addressing my concerns.	0.844	−0.727 (0.100)	−0.179 (0.199)

**Factor 3** Risk Perception for Vaccine Passport Eigen-values 2.930; Variance Explained 23.394%; Cronbach’s alpha 0.966			
1. The thought of launching a vaccine passport/certificate makes me feel anxious.	−0.877	1.664 (0.100)	1.925 (0.199)
2. The thought of traveling with a vaccine passport/certificate makes me feel psychologically uncomfortable.	−0.865	1.479 (0.100)	1.255 (0.199)
3. Traveling with a vaccine passport/certificate is risky.	−0.882	1.608 (0.100)	1.697 (0.199)
4. Traveling with a vaccine passport/certificate creates a higher chance of getting the COVID-19.	−0.877	1.589 (0.100)	1.714 (0.199)
5. I worry about paying extra fees when traveling with a vaccine passport/certificate.	−0.865	1.567 (0.100)	1.561 (0.199)
6. I am concerned that traveling with a vaccine passport/certificate will incur additional unexpected costs.	−0.878	1.584 (0.100)	1.540 (0.199)

**Factor 4** Perception of Incentives Eigen-values 13.224; Variance Explained 28.225%; Cronbach’s alpha 0.965			
1. It is tempting that hotels offer room discounts for vaccine passport/certificate holders.	0.781	−0.863 (0.100)	0.230 (0.199)
2. It is tempting that some hotels offer free beverage for vaccine passport/certificate holders.	0.797	−0.552 (0.100)	−0.446 (0.199)
3. It is tempting that the airline companies offer airline ticket discounts for vaccine passport/certificate holders.	0.821	−0.721 (0.100)	−0.255 (0.199)
4. It is tempting that the airline companies offer ticket upgrade (first class, business class etc.) events for vaccine passport/certificate holders.	0.801	−0.674 (0.100)	−0.271 (0.199)
5. It is tempting that the restaurants offer discounts events for vaccine passport/certificate holders.	0.807	−0.813 (0.100)	−0.015 (0.199)
6. It is tempting that the restaurants offer a variety of events for vaccine passport/certificate holders.	0.811	−0.689 (0.100)	−0.304 (0.199)
7. It is tempting that the tourism attractions offer free ticket events for vaccine passport/certificate holders.	0.811	−0.730 (0.100)	−0.192 (0.199)
8. It is tempting that tourism attractions offer a variety of events for vaccine passport/certificate holders.	0.799	−0.792 (0.100)	−0.105 (0.199)
KMO and Bartlett’s test = 0.966, Significance = 0.000; Total Variance Explained 81.916%			

### Confirmatory Factor Analysis of the Measurement Model

In Tables 3, 4, a confirmatory factor analysis (CFA) was applied to confirm the factor structure explored in the EFA. The final results of the CFA reported an adequate and satisfactory score for the overall fit indices (*X*^2^ = 1669.188, *df* = 751, *X*^2^*/df* = 2.223, *p* < 0.001, RMSEA = 0.045, CFI = 0.963, IFI = 0.963, and TLI = 0.960.). The standardized factor loading values (Beta coefficient) of each item has exceeded the proposed threshold of 0.7. All average variance extracted (AVE) scores and construct reliability values (CR) were also >0.6 and 0.85, respectively, thus absolutely supporting and emphasizing the convergent validity.

**TABLE 3:** CFA results.

Factors	Factor loading	Mean
**Factor 1** Perceived Usefulness of Vaccine Passport		
1. It is convenient to use when the vaccine passport/certificate comes out as a paper or digital format.	0.889	5.160
2. Using vaccine passport/certificate when traveling is helpful as it reduces separate testing for COVID-19.	0.843	4.940
3. Vaccine passport/certificate gives me faster access to travel without a two-week quarantine.	0.869	5.040
4. Vaccine passport/certificate use improves the quality of travel.	0.896	5.020
5. Vaccine passport/certificate will be useful during traveling.	0.850	5.100
6. Using the vaccine passport/certificate will be fit my needs.	0.916	5.070

**Factor 2** Destination trust for vaccine passport		
1. I believe that destinations that accept vaccinated travelers will provide high quality and efficient tourism services.	0.877	4.990
2. I can rely on destinations that accept travelers with vaccine passport/certificate.	0.847	4.970
3. I believe that destinations that accept vaccine passport/certificate holders will be honest and sincere in addressing my concerns.	0.786	4.960

**Factor 3** Risk perception for vaccine passport		
1. The thought of launching a vaccine passport/certificate makes me feel anxious.	0.911	2.520
2. The thought of traveling with a vaccine passport/certificate makes me feel psychologically uncomfortable.	0.918	2.370
3. Traveling with a vaccine passport/certificate is risky.	0.917	2.440
4. Traveling with a vaccine passport/certificate creates a higher chance of getting the COVID-19.	0.910	2.370
5. I worry about paying extra fees when traveling with a vaccine passport/certificate.	0.910	2.450
6. I am concerned that traveling with a vaccine passport/certificate will incur additional unexpected costs.	0.889	2.400

**Factor 4** Perception of incentives		
1. It is tempting that hotels offer room discounts for vaccine passport/certificate holders.	0.879	5.020
2. It is tempting that some hotels offer free beverage for vaccine passport/certificate holders.	0.861	4.730
3. It is tempting that the airline companies offer airline ticket discounts for vaccine passport/certificate holders.	0.886	4.940
4. It is tempting that the airline companies offer ticket upgrade (first class, business class etc.) events for vaccine passport/certificate holders.	0.877	4.920
5. It is tempting that the restaurants offer discounts events for vaccine passport/certificate holders.	0.873	4.950
6. It is tempting that the restaurants offer a variety of events for vaccine passport/certificate holders.	0.875	4.790
7. It is tempting that the tourism attractions offer free ticket events for vaccine passport/certificate holders.	0.892	4.970
8. It is tempting that tourism attractions offer a variety of events for vaccine passport/certificate holders.	0.894	4.920

**Factor 5** Attitude toward the use of vaccine passport for travel abroad		
1. For me, traveling abroad with a vaccine passport/certificate is a positive/negative thing.	0.898	4.780
2. For me, traveling abroad with a vaccine passport/certificate is a wise/senseless thing.	0.876	4.590
3. For me, traveling abroad with a vaccine passport/certificate is a beneficial/harmful thing.	0.882	4.680
4. For me, traveling abroad with a vaccine passport/certificate is a pleasant/unpleasant thing.	0.886	4.700
5. For me, traveling abroad with a vaccine passport/certificate is an extremely desirable/undesirable.	0.892	4.770

**Factor 6** Intention to travel abroad		
1. I am willing to travel abroad, once a vaccine passport/certificate is issued	0.883	4.440
2. I prefer to travel abroad with a vaccine passport/certificate during COVID-19 period.	0.852	4.170
3. I plan to travel abroad, once a vaccine passport/certificate is issued.	0.892	4.250
4. If a vaccine passport/certificate is available, I will travel abroad within 12 months.	0.871	4.180
5. I want to travel abroad without quarantine for 2 weeks with a vaccine passport/certificate.	0.881	4.470

**Factor 7** Intention to travel abroad		
1. For me, traveling domestically with a vaccine passport/certificate is a positive/negative thing.	0.824	5.280
2. For me, traveling domestically with a vaccine passport/certificate is a wise/senseless thing.	0.821	5.210
3. For me, traveling domestically with a vaccine passport/certificate is a beneficial/harmful thing.	0.801	5.300
4. For me, traveling domestically with a vaccine passport/certificate is a pleasant/unpleasant thing.	0.807	5.210
5. For me, traveling domestically with a vaccine passport/certificate is an extremely desirable/undesirable thing.	0.822	5.150

**Factor 8** Intention to travel domestically		
1. I prefer to travel domestically with a vaccine passport/certificate during COVID-19 period.	0.858	5.190
2. I plan to travel domestically, once a vaccine passport/certificate is issued.	0.806	5.240
3. If a vaccine passport/certificate is available, I will travel domestically within 12 months.	0.783	5.320

**TABLE 4:** Results of measurement model and correlations.

	INTABR	PUFVP	DTVP	RPVP	PINC	ATTABR	ATTDOM	INTDOM	CR	AVE
INTABR	0.876								0.943	0.767
PUFVP	0.282	0.878							0.953	0.77
DTVP	0.288	0.683	0.838						0.876	0.701
RPVP	–0.127	–0.576	–0.433	0.909					0.966	0.827
PINC	0.344	0.796	0.587	–0.53	0.88				0.965	0.774
ATTABR	0.645	0.552	0.512	–0.383	0.539	0.887			0.948	0.786
ATTDOM	0.313	0.645	0.435	–0.459	0.656	0.521	0.815		0.908	0.664
INTDOM	0.237	0.305	0.296	–0.183	0.316	0.355	0.535	0.816	0.857	0.666

*Goodness-of-Fit Statistics:χ^2^ = 1669.188, df = 751, χ^2^/df = 2.223, p < 0.001, RMSEA = 0.045, CFI = 0.963, IFI = 0.963, and TLI = 0.960.*

*CR, Composite reliability; AVE, Average variance extracted; PUFVP, Perceived Usefulness of Vaccine Passport; DTVP, Destination Trust for Vaccine Passport; RPVP, Risk Perception for Vaccine Passport; PINC, Perception of Incentives; ATTABR, Attitude toward the Use of Vaccine Passport for travel abroad; ATTDOM, Attitude toward the Use of Vaccine Passport for domestic travel; INTABR, Intention to travel abroad; INTDOM, Intention to travel domestically.*

### Structural Equation Modeling

**FIGURE 1:**
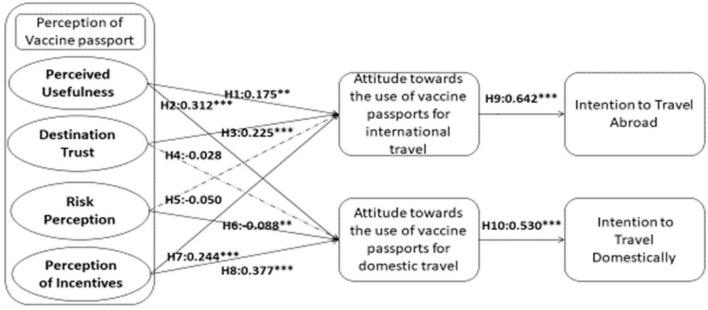
Results of hypotheses tests. **P < 0.05 and ***P < 0.01.

As exhibited in Table 5 and Figure 1, the overall fit indices findings of the structural equation modeling (SEM) determine a satisfactory level of fit (X^2^ = 1734.158, *df* = 763, X^2^/*df* = 2.273, *p* < 0.001, RMSEA = 0.046, CFI = 0.961, IFI = 0.961, and TLI = 0.958.). We evaluated a total of 10 direct relationships in this study, among them, the results reported 8 out of 10 as supported hypotheses. PUFVP has significant positive effects on ATTABR and ATTDOM (H1: β = 0.175, *t* = 2.397, *p* < 0.05; H2: β = 0.312, *t* = 4.475, *p* < 0.01), and PINC has positive effects on ATTABR and ATTDOM (H7: β = 0.244, *t* = 3.928, *p* < 0.01; H8: β = 0.377, *t* = 6.271, *p* < 0.01). However, DTVP has positive effects on ATTABR while it does not have any effects on ATTDOM (H3: β = 0.225, *t* = 4.161, *p* < 0.01; H4: not supported), and RPVP has negative effects on ATTDOM while it does not have any effects on ATTABR (H6: β = −0.088, *t* = −2.122, *p* < 0.05; H5: not supported). Lastly, ATTABR has positive effects on INTABR and ATTDOM has positive effects on INTDOM (H9: β = 0.642, *t* = 16.696, *p* < 0.01; H10: β = 0.530, *t* = 11.826, *p* < 0.01).

**TABLE 5:** Results of SEM.

Hypotheses	Paths	Coefficients	*t-*values	Results
H1: PUFVP	→ATTABR	0.175	2.397**	H1: Supported
H2: PUFVP	→ATTDOM	0.312	4.475***	H2: Supported
H3: DTVP	→ATTABR	0.225	4.161***	H3: Supported
H4: DTVP	→ATTDOM	–0.028	–0.550	H4: Not supported
H5: RPVP	→ATTABR	–0.050	–1.148	H5: Not supported
H6: RPVP	→ATTDOM	–0.088	−2.122**	H6: Supported
H7: PINC	→ATTABR	0.244	3.928***	H7: Supported
H8: PINC	→ATTDOM	0.377	6.271***	H8: Supported
H9: ATTABR	→INTABR	0.642	16.696***	H9: Supported
H10: ATTDOM	→INTDOM	0.530	11.826***	H10: Supported

*Goodness-of-Fit Statistics: χ^2^ = 1734.158, df = 763, χ^2^/df = 2.273, p < 0.001, RMSEA = 0.046, CFI = 0.961, IFI = 0.961, and TLI = 0.958.*

**P < 0.05, ***P < 0.01. PUFVP, Perceived Usefulness of Vaccine Passport; DTVP, Destination Trust for Vaccine Passport; RPVP, Risk Perception for Vaccine Passport; PINC, Perception of Incentives; ATTABR, Attitude toward the Use of Vaccine Passport for travel abroad; ATTDOM, Attitude toward the Use of Vaccine Passport for domestic travel; INTABR, Intention to travel abroad; INTDOM, Intention to travel domestically.

### Results of Means Comparisons: Gender With Attitude and Travel Intention

**FIGURE 2:**
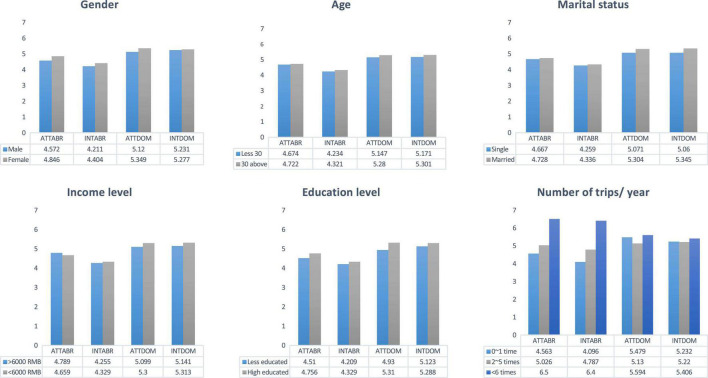
Means comparison results by demographics.

The independent sample *t*-tests of respondents found significant differences in tourists’ attitude toward traveling abroad and intention to travel abroad across two gender groups (attitude toward travel abroad: *F* = 6.552, *p* = 0.011; intention to travel abroad: *F* = 5.951, *p* = 0.015). However, in tourists’ attitude toward domestic travel, it has significant differences between male and female but to the intention to domestic travel has no differences by gender (attitude toward travel domestically: *F* = 6.332, *p* = 0.012; intention to travel domestically: *F* = 0.195, *p* = 0.659) (Table 6 and Figure 2). As for the mean score, females had a higher attitude toward both overseas and domestic travel than males, and their intention to travel abroad and domestically was higher than that of males. These results confirmed that the female group had a stronger desire for travel than the male group (ATTABR: *M*_male_ = −4.572 Vs. *M*_female_ = 4.846; INTABR: *M*_male_ = 4.211 Vs. *M*_female_ = 4.404; ATTDOM: *M*_male_ = 5.12 Vs. *M*_female_ = 5.349; INTDOM: *M*_male_ = 5.231 Vs. *M*_female_ = 5.277) (Table 6).

**TABLE 6:** *T*-test results.

**Results of *t*-test (gender)**						
**Variables**	**Gender**	**N**	***F*-value**	***p*-value**	**Mean**	***SD***

ATTABR	Male	312	6.552	0.011**	4.572	1.504
	Female	289			4.846	1.359
INTABR	Male	312	5.951	0.015**	4.211	1.553
	Female	289			4.404	1.412
ATTDOM	Male	312	6.332	0.012**	5.12	1.374
	Female	289			5.349	1.183
INTDOM	Male	312	0.195	0.659	5.231	1.273
	Female	289			5.277	1.254

**Results of *t*-test (age)**						

**Variables**	**Age**	***N***	***F*-value**	***p*-value**	**Mean**	**SD**

ATTABR	Less 30	225	4.474	0.035**	4.674	1.349
	30 above	376			4.722	1.496
INTABR	Less 30	225	5.366	0.021**	4.234	1.377
	30 above	376			4.321	1.554
ATTDOM	Less 30	225	3.067	0.080	5.147	1.367
	30 above	376			5.280	1.240
INTDOM	Less 30	225	1.635	0.201	5.171	1.316
	30 above	376			5.301	1.230

**Results of *t*-test (marital status)**						

**Variables**	**Marital status**	**N**	***F-*value**	***p*-value**	**Mean**	**SD**

ATTABR	Single	196	3.118	0.078	4.667	1.359
	Married	399			4.728	1.478
INTABR	Single	196	0.980	0.323	4.259	1.434
	Married	399			4.336	1.517
ATTDOM	Single	196	1.911	0.167	5.071	1.370
	Married	399			5.304	1.248
INTDOM	Single	196	3.346	0.068	5.060	1.363
	Married	399			5.345	1.206

**Results of *t*-test (income level)**						

**Variables**	**Income level**	***N***	***F*-value**	***p*-value**	**Mean**	**SD**

ATTABR	Under 6000	210	2.238	0.135	4.789	1.374
	6000 above	391			4.659	1.476
INTABR	Under 6000	210	0.184	0.668	4.255	1.467
	6000 above	391			4.329	1.504
ATTDOM	Under 6000	210	0.101	0.750	5.099	1.292
	6000 above	391			5.300	1.284
INTDOM	Under 6000	210	0.382	0.537	5.141	1.274
	6000 above	391			5.313	1.255

**Results of *t*-test (Education level)**						

**Variables**	**Education level**	***N***	***F*-value**	***p*-value**	**Mean**	**SD**

ATTABR	Less educated	127	0.000	0.984	4.510	1.423
	High educated	474			4.756	1.444
INTABR	Less educated	127	0.003	0.960	4.209	1.489
	High educated	474			4.329	1.491
ATTDOM	Less educated	127	3.914	0.048**	4.930	1.374
	High educated	474			5.310	1.255
INTDOM	Less educated	127	0.198	0.657	5.123	1.283
	High educated	474			5.288	1.257

**P < 0.05 and ***P < 0.01.

### Results of Means Comparisons: Age With Attitude and Travel Intention

The findings of the *t*-tests revealed that in tourists’ attitude toward traveling abroad and intention to travel abroad with different age levels (attitude toward travel abroad: *F* = 4.474, *p* = 0.035; intention to travel abroad: *F* = 5.366, *p* = 0.021), while in attitude toward domestic travel and intention to domestic travel have no differences with different age levels (attitude toward travel domestically: *F* = 3.067, *p* = 0.080; intention to travel domestically: *F* = 1.635, *p* = 0.201) (Table 6 and Figure 2).

### Results of Means Comparisons: Marital Status, Income Level, and Education Level With Attitude and Travel Intention

As results of the independent sample *t*-tests for marital status and income level, it was found that attitude toward both domestic and international travel and intention to travel both abroad and domestically have no differences (Marital status, ATTABR: *F* = 3.118, *p* = 0.078;INTABR: *F* = 0.980, *p* = 0.323; ATTDOM: *F* = 1.911, *p* = 0.167; INTDOM: *F* = 3.346, *p* = 0.068; Income level, ATTABR: *F* = 2.238, *p* = 0.135;INTABR: *F* = 0.184, *p* = 0.668; ATTDOM: *F* = 0.101, *p* = 0.750; INTDOM: *F* = 0.382, *p* = 0.537) (Table 6 and Figure 2).

The level of education is divided into two groups: less educated and highly educated. The *t*-tests on academic background showed that attitude toward traveling abroad and intention to travel both abroad and domestically have no differences across the two groups, while in attitude toward domestic travel had significant difference between two groups (ATTABR: *F* = 0.000, *p* = 0.984; INTABR: *F* = 0.003, *p* = 0.960; ATTDOM: *F* = 3.914, *p* = 0.048; INTDOM: *F* = 0.198, *p* = 0.657) (Table 6 and Figure 2).

### Results of Means Comparisons: Number of Trips per Year With Attitude and Travel Intention

The ANOVA tests (Table 7 and Figure 2) revealed significant differences in attitude toward traveling abroad and intention to travel abroad across number of trips per year (ATTABR: *F* = 8.102, *p* = 0.000; INTABR: *F* = 16.051, *p* = 0.000). However, attitude toward domestic travel had differences across three groups, while intention to travel domestically did not vary by the same groups (ATTABR: *F* = 6.316, *p* = 0.002; INTABR: *F* = 0.940, *p* = 0.391). As the mean scores shown, those who travel frequently a year showed higher attitudes and intention to travel than those who travel less in a year (ATTABR: *M*_0–1_ = 4.563Vs. *M*_2–5_ = 5.026 Vs. *M*_6a*bove*_ = 6.500; INTABR: *M*_0–1_ = 4.096 Vs. *M*_2–5_ = 4.787 Vs. *M*_6a*bove*_ = 6.400; ATTDOM: *M*_0–1_ = 5.479Vs. *M*_2–5_ = 5.130 Vs. *M*_6a*bove*_ = 5.594; INTDOM: *M*_0–1_ = 5.232Vs. *M*_2–5_ = 5.220 Vs. *M*_6a*bove*_ = 5.406).

**TABLE 7:** Results of ANOVA (number of trips/year).

Variables	Number of trips per year	*N*	*F*-value	*p*-value	Mean	*SD*
ATTABR	0∼1	425	8.102	0.000***	4.563	1.420
	2∼5	174			5.026	1.441
	6 or above	2			6.500	0.141
INTABR	0∼1	425	16.051	0.000***	4.096	1.452
	2∼5	174			4.787	1.464
	6 or above	2			6.400	0.283
ATTDOM	0∼1	33	6.316	0.002***	5.479	1.095
	2∼5	463			5.130	1.308
	6 or above	105			5.594	1.193
INTDOM	0∼1	33	0.940	0.391	5.232	1.303
	2∼5	463			5.220	1.268
	6 or above	105			5.406	1.233

**P < 0.05 and ***P < 0.01.

## Discussion and Implications

In this study, we investigated how travelers think about the use of vaccine passports. The use of vaccine passports is the way back to normal. The purpose of needing a vaccine passport is to facilitate accessibility, mobility, participate in large events, visiting public places, and return individuals to face-to-face work (Brazal, 2021). This empirical study expanded knowledge of tourists’ attitudes toward travel and the formation of travel intentions using demographics and perceptions of use of vaccine passports by tourists (Zopiatis et al., 2021). Expand understanding of the demand of Chinese tourists and provide helpful resources for business and research on the Chinese market.

The SEM results revealed that the perceived usefulness of vaccine passports has significant positive effects on attitude toward travel abroad and attitude toward domestic travel. This finding is slightly consistent with previous research results (Rivera et al., 2015; Upadhyay et al., 2018). This result means that traveler expectations for vaccine passports are significant. In addition, vaccine incentives offered by the tourism sector have positive effects on attitude toward traveling abroad and attitude toward domestic travel. This result suggests that incentives provided by the tourism industry to encourage vaccinated people were found to have a positive effect on the attitudes of both domestic and international travelers. Furthermore, these incentives affect the image and brand value of the tourism industry, thereby increasing the loyalty of tourists to the tourism industry and maintaining a close relationship during the pandemic. However, destination trust has positive effects on attitude toward travel abroad but not to attitude toward domestic travel. This is almost in line with the results of previous studies on attitudes toward international travel, but it does not seem to have affected the trust of domestic destinations because travelers are well-aware of the domestic COVID-19 situation. Therefore, it was concluded that travelers are more positive about using vaccine passports when traveling abroad. When it comes to risk perception, it has negative effects on attitude toward domestic travel but not to attitude toward travel abroad. During domestic travel, it can be seen that the vaccine passports play a positive psychological, safety and financial role for travelers, alleviating risk perception. Lastly, the attitude is likely to have a positive influence on both domestic and international travel.

In addition, as a result of the mean comparison analysis according to gender, females had higher travel desires than the male group, and by age, the elderly group had a more positive attitude toward the use of vaccine passports. In addition, according to the number of annual travels, the group who travels frequently showed a more positive attitude toward travel with a vaccine passport. The mean comparison analysis result is expected to help launch strategic travel products for a specific target.

Based on these results, some theoretical and managerial suggestions are presented. First, Vaccine passports are used by individuals, so it is very important for academia to recognize individuals’ perception about vaccine passports. However, in many previous studies, research on the travel industry aspects were mainly conducted, and there were very few studies on consumers’ awareness of vaccine passport use. Therefore, this study contributed to filling the literature gap on consumer perception during the COVID-19 period. Second, this study materialized the public’s perception of vaccine passports through prior research and in-depth interviews, and it is expected that this understanding can provide informative information to the travel industry academia. However, vaccine passports require access to personal medical information, so data protection should be tackled by tourism industry. Third, in previous studies, travel intentions were not divided into domestic and overseas. However, in this study, through an empirical study, factors affecting domestic travel intention and international travel intentions were analyzed in detail. These findings from this study provide a better understanding of traveler behavior and a theoretical contribution to tourism industry research. Although many people’s daily lives are being disrupted by COVID-19, people’s attitudes toward the usefulness and incentives of vaccine passports are positive, and many people still want to travel domestically and internationally. In particular, the use of vaccine passports will be played a big role for international students and certain groups who need to travel on business. Vaccine passports have a positive effect on people’s attitudes toward destination trust and alleviate people’s awareness of financial and health risks. Moreover, a positive attitude is a consideration for marketers in the tourism industry looking to increase tourist visits (Hasan et al., 2020). Therefore, government and travel industry managers should encourage more people to actively use vaccine passports to save the travel industry. In addition, online travel agencies should cooperate with travel industry managers to provide more information through online resources to customers purchasing products by accurately stating whether vaccine passports are required/not required, incentive items, self-quarantine required/not required, and other items to prepare. The only way to save the travel industry is to cooperate with the government and all travel industries to create a safe and comfortable travel space. Vaccine passports are used as a tool of travel (Lee et al., 2022), so safety policies must be followed by each industry. For example, airlines, hotels, restaurants, etc., must secure social distance between customers and customers for a more comfortable trip, strive for cleanliness and disinfection, and provide customers with information about nearby hospitals and public health centers.

### Limitations and Future Study

Although this study showed both theoretical and managerial implications, there are some limitations that can suggest a roadmap for future research design. First, this study was conducted and implemented at a critical time in severe virus spread (e.g., the delta variation). Therefore, it seems that better results can be obtained if the study is conducted once again after the delta and new (Omicron) virus have been alleviated. Second, this study only targeted the Chinese market. It is difficult to generalize the results of these studies as China currently strictly enforces overseas entry and exit. Therefore, conducting research with this frame in other countries or cities helps to generalize this study. Third, this study used a convenient sampling method, so the study subjects were limited to relatively young people. Considering this benefit in future studies, it is also necessary to examine the awareness of the use of vaccine passports in elderly people. Additionally, examining people’s varying perceptions of the use of vaccine passports by the country could significantly increase the impact on the travel industry. Lastly, in future research, we hope that the mean comparisons in demographic characteristics will also be applied to the four factors for vaccine passport perception. It is predicted that the research results will help to understand the characteristics of consumers in detail and establish marketing strategies for market segmentation.

## Data Availability Statement

The raw data supporting the conclusions of this article will be made available by the authors, without undue reservation.

## Author Contributions

All authors contributed to conceptualization, formal analysis, investigation, methodology, and writing and editing the original draft.

## Conflict of Interest

The authors declare that the research was conducted in the absence of any commercial or financial relationships that could be construed as a potential conflict of interest.

## Publisher’s Note

All claims expressed in this article are solely those of the authors and do not necessarily represent those of their affiliated organizations, or those of the publisher, the editors and the reviewers. Any product that may be evaluated in this article, or claim that may be made by its manufacturer, is not guaranteed or endorsed by the publisher.
